# Detection of Influenza D Virus using Reverse Transcription Loop-Mediated Isothermal Amplification

**DOI:** 10.3201/eid3210.260499

**Published:** 2026-10

**Authors:** Carlos Abelardo dos Santos, Jialu Li, Pauline M. van Diemen, Andrew McMahon, Benjamin C. Mollett, Andrew M. Ramsay, Meshach M. Maina, Joe James, Helen E. Everett, Janet M. Daly, Nicole C. Robb

**Affiliations:** Warwick Medical School, University of Warwick, Coventry, UK (C.A. dos Santos, J. Li, N.C. Robb); Animal and Plant Health Agency Weybridge, Addlestone, UK (P.M. van Diemen, B.C. Mollett, A.M. Ramsay, J. James, H.E. Everett); School of Life Sciences, University of Warwick, Coventry (A. McMahon); One Virology, Wolfson Centre for Global Virus Research, University of Nottingham, Nottingham, UK (M.M. Maina, J.M. Daly)

**Keywords:** influenza, viruses, zoonoses, influenza D, RT-LAMP, molecular diagnostics, United Kingdom

## Abstract

The *Orthomyxoviridae* family includes influenza D virus (IDV), an emerging pathogen primarily affecting cattle and swine; there is evidence of cross-species transmission and potential zoonotic risk. Although active human infections have yet to be confirmed, high seroprevalence in cattle-exposed populations highlights the need for continued surveillance. We developed and validated a rapid, field-deployable reverse transcription loop-mediated isothermal amplification assay for IDV detection; specificity was 99.2% and sensitivity ranged from 95.6% (cycle quantification <30) to 81.8% (cycle quantification <40). This method offers a cost-effective, accessible alternative to quantitative reverse transcription PCR, enabling improved monitoring of IDV and reinforcing preparedness for emerging influenza threats.

The family *Orthomyxoviridae* consists of 9 genera, 4 of which are influenza viruses. Influenza A virus (IAV) and influenza B virus (IBV) are the main viruses responsible for human seasonal influenza epidemics; IAV poses a pandemic threat linked to bidirectional transmission between animals and humans. Both IAV and IBV can cause severe illness in humans, whereas influenza C virus (ICV) infection is associated with milder upper respiratory tract symptoms, most commonly in children < 2 years of age. Influenza D virus (IDV) was first discovered in 2011 in pigs in Oklahoma, USA, and was subsequently found in cattle, which are now considered the main livestock reservoir. In cattle, IDV infection is associated with bovine respiratory disease as the primary viral infection, which might predispose cattle to opportunistic secondary bacterial infections (*1*).

Although most IDV occurrences have been in swine and cattle, IDV antibodies have also been found in other domesticated animals, such as sheep (*2*), goats (*3*), horses (*3*), wild boars (*4*), and camelids (*5*), suggesting that cross-species transmission might occur. Mice, ferrets, and guinea pigs have also been found to be susceptible to experimental viral infection (*6*). Similarly to ICV, IDV has been shown to bind to sialic acid receptors on the cell surface of the host, specifically 9-O-acetylated sialic acid receptors (*7*). Those receptors are found throughout the respiratory tract in cattle, as well as in the nasal and pharyngeal epithelium of pigs, sheep, goats, and horses, suggestive of a potentially wide host range (*8*).

IDV has been shown to propagate effectively in various human cell types (*9*), but no active infection in humans has been reported to date; although IDV was detected in a nasal wash sample from a swine farm worker in Malaysia, no infectious virions were retrieved (*10*). The presence of IDV antibodies has been the primary method of inferring past exposure to IDV in humans. A 2011 study (*11*) found a 1.3% seroprevalence of IDV antibodies in the general human population in the United States and Canada. Another study in Italy discovered that the seroprevalence of IDV in the general human population increased from 5% in 2005 to 46% in 2014 (*12*). In human cohorts occupationally exposed to cattle, the prevalence of IDV antibodies reached as high as 97% (*13*), reflecting a similar prevalence of antibodies in cattle (*14*).

Although IDV infection has not been observed to induce severe disease in humans, monitoring its prevalence remains crucial because of the ability of influenza viruses to evolve and transmit across species barriers. Antibody detection serves as a robust epidemiologic tool on a population scale (*15*), but quantitative reverse transcription PCR (qRT-PCR) is the standard for detecting viral RNA (and thus active infection) at an individual level. However, the need for specialized laboratories and trained personnel to process and analyze samples, coupled with high costs and limited utility for field-based testing, poses a substantial challenge to high throughput. Reverse transcription loop-mediated isothermal amplification (RT-LAMP) is a rapid, highly sensitive, and cost-effective molecular diagnostic tool that has emerged as a potential alternative to PCR-based methods (*16*). RT-LAMP can be performed at a constant temperature and return a result in <1 hour, making it well suited for point-of-sample testing. The success of RT-LAMP during the COVID-19 pandemic underscores its potential as a valuable tool in our ongoing efforts to combat infectious diseases (*17*). We report an RT-LAMP assay specifically designed to detect IDV with particular application for field-setting diagnostic assays in resource-poor settings. 

## Materials and Methods

### Study Samples

We tested a total of 28 experimentally spiked samples and 46 isolated RNA samples from swine and cattle using the RT-LAMP assay. The animal samples consisted of nasal swab and respiratory tissue samples (lung, viscera) from pigs and cattle with signs of respiratory disease (cough and/or dyspnea) submitted through the passive Animal and Plant Health Agency surveillance networks and contained a mixture of samples positive or negative for IAV or IDV.

### Virus Isolates

We propagated the isolates D/swine/Oklahoma/1334/2011 (*11*) in embryonated chicken eggs and D/bovine/France/5920/2014 (*18*,*19*) on hRT18G cells. Both strains are well characterized and produced with a minimum number of passages to avoid loss of strain fidelity. D/swine/England/126471/2023 is a more recent isolate and was propagated on ST cells (*20*).

### RNA Extraction

We carried out preisolation steps depending on the nature of the sample. For viral isolates, we lysed 20 μL of the cell supernatant with 350 μL of Monarch StabiLyse DNA/RNA Buffer (New England Biolabs, https://www.neb.com) and carried out the extraction according to the manufacturer’s protocol. We also extracted RNA from a swine lung fragment by excising a 10 mg tissue fragment and lysing it in 200 μL of StabiLyse DNA/RNA Buffer and 200 μL of nuclease-free water (IDT, https://eu.idtdna.com) in a FastPrep-24 bead beater homogenizer (MP Biomedicals, https://www.mpbio.com) for ≈10 minutes. We extracted total RNA from each preparation using the Monarch Spin RNA Isolation Kit (New England Biolabs), according to the manufacturer’s instructions. We extracted positive (IDV cultures) and negative (with nuclease-free water) controls with every batch and tested them by qRT-PCR to guarantee extraction efficiency and the absence of cross-contamination during extraction.

### qRT-PCR for IDV

The qRT-PCR primers and probes for IDV detection used in this study were originally published by Faccini et al. (*21*). We performed the reactions in a 384-well Quant Studio 5 Real-Time PCR System (Thermo Fisher Scientific, https://www.thermofisher.com) using the Luna Universal Probe One-Step RT-qPCR Kit (New England Biolabs), according to the manufacturer’s instructions. The primers and probe target the polymerase basic (PB) 1 gene (from position 1215 to 1323; GenBank accession no. JQ922306); we designed a PB1 gBlock to use as control containing the nucleotides from position 500 to 1,500. The gBlock was synthesized by Integrated DNA Technologies (https://www.idtdna.com). We eluted the gBlock according to the manufacturer’s protocol and confirmed the concentration by UV absorbance using a NanoDrop spectrophotometer (Thermo Fisher Scientific). We used serial dilutions ranging from 10^6^ to 10 copies of the IDV gBlock to generate a standard curve, which we used to quantify the RNA material from isolates, mock samples, and samples.

### RT-LAMP Primer Design

We designed primers targeting the 2 most conserved genes in IDV, PB1 and PB2 (*18*,*22*) (Appendix Table 1). We downloaded all available sequences from the National Center for Biotechnology Information (NCBI) Nucleotide database (https://www.ncbi.nlm.nih.gov/nucleotide) and generated alignments using the MAFFT multiple sequence alignment tool (https://mafft.cbrc.jp/alignment/software). We designed the primers for RT-LAMP against PB1 and PB2 using the PrimerExplorer version 5 software (https://primerexplorer.eiken.co.jp/lampv5e/index.html). To improve coverage and guarantee amplification of the maximum number of sequences, we set the consensus threshold to 90%. We marked all mutated positions in the primer design software and created the primers using the common design option, which enables primers to be designed targeting mutated regions if the position of the variable nucleotide within the primer is unlikely to affect amplification efficiency. We filtered primers by end stability and set the maximum ΔG for the 3′ end of F3/B3, F2/B2, LF/LB and the maximum ΔG for the 5′ end of F1c/B1c to be <–4.00 kcal/mol. We also checked primers for the presence of stable hairpins, self-dimers, and homodimers using IDT’s Oligo Analyzer tool (https://eu.idtdna.com/pages/tools/oligoanalyzer). We did not synthesize sets containing primers with stable hairpins or dimers. The primers FIP and BIP consist of 2 different regions (F1c/F2 for FIP and B1c/B2 for BIP), which mediate the creation of the loops characteristic of LAMP. Target recognition by LAMP primers creates an ever-growing concatemer containing single-stranded loops that also serves as a target for continuous amplification. To decrease local Tm in the loops and lower the chances of undesired secondary structures that could hinder amplification, we inserted T linkers (*23*) between the F1c/F2 and B1c/B2 regions in the FIP/BIP primers. We also aligned primers against other influenza viruses to guarantee specificity for influenza D and followed that with a BLAST analysis (https://blast.ncbi.nlm.nih.gov).

### Colorimetric One-Step RT-LAMP Assay for IDV

We prepared all reactions as a total volume of 20 μL, each containing a final concentration of 1X WarmStart Colorimetric LAMP Master Mix with UDG (Uracil DNA Glycosylate) (New England Biolabs), 1× primer mix (containing 0.2 μM F3/B3, 1.6 μM FIP/BIP and 0.8 μM LF/LB primers), 10% (vol/vol) of target, and nuclease-free water (Integrated DNA Technologies). For real-time analysis, we added 1 μM of SYTO9 (Thermo Fisher Scientific) to the reaction mixture. To optimize the assay, we incubated the reactions in a QuantStudio 3 real-time thermocycler (Thermo Fisher Scientific) at 65°C for 80 cycles, each cycle consisting of 45 seconds of incubation followed by fluorescence read in the FAM channel for 15 seconds. We incubated subsequent reactions in a conventional T-100 PCR instrument (Bio-Rad Laboratories, https://www.bio-rad.com) at 65°C for 60 minutes. Unless otherwise specified, we performed all reactions in triplicate.

### Sensitivity and Specificity of IDV Assay

To assess whether we could differentiate IDV from closely related viruses, we tested the RT-LAMP assay for IDV against gBlocks containing the PB2 target gene segment of all other influenza strains. We used the gBlock containing the complete PB2 gene for IDV (D/swine/Oklahoma/1334/2011) as a positive control in our reactions and used gBlocks containing the complete PB2 gene for IAV (A/Puerto Rico/8/1934), IBV (B/Lee/1940), and ICV (C/Ann Arbor/1/50) as negative controls. We carried out reactions using either 2 μL of the respective gBlock controls or the equivalent amount of nuclease-free water as a nontemplate control (NTC). All gBlocks were tested at a concentration of 10^6^ copies per μL for 1 hour. We analyzed results in triplicate on the basis of the colorimetric change in the reaction from pink (negative) to yellow (positive).

To determine the sensitivity of the assay, we mixed equimolar amounts of extracted RNA from each of the 3 viral isolates (D/bovine/France/5920/2014, D/swine/Oklahoma/1334/2011, and D/swine/England/126471/2023), which had been quantified by qRT-PCR using the assay described previously. We serially-diluted the RNA mix and verified the concentrations by qRT-PCR. We tested the serially diluted RNA mix by RT-LAMP in octuplicate and fitted a Probit regression curve (dose-response plot) using SPSS Statistics 29.0.2.0 (IBM, https://www.ibm.com). We defined the limit of detection to be equal to the minimum number of copies detected in >95% of the reactions.

We analyzed the amplification products on a 2% agarose gel in 1X TBE (Tris-Borate-EDTA) Buffer using SYBR Safe DNA Gel Stain (Thermo Fisher Scientific) and the Quick-Load 100 bp DNA Ladder (New England Biolabs). We ran the gel at 100V for 60 minutes and acquired images with a ChemiDoc Imaging System (BioRad).

### Evaluation Using Spiked and Field-Collected Animal Samples

To assess the performance of the test in a more complex sample matrix, we prepared 28 spiked samples by mixing total purified RNA extracted from swine lung tissue with variable amounts of viral RNA from the 3 virus isolates used in this study. We quantified samples by qRT-PCR and tested by RT-LAMP. We analyzed results by the color change from pink (negative) to yellow (positive) in the reaction after 60 minutes of incubation. We assessed the specificity and sensitivity of the RT-LAMP assay for detecting IDV by comparing its results with those of the qRT-PCR using the free online tool Medcalc (https://www.medcalc.org/calc/diagnostic_test.php).

## Results

### Optimization of RT-LAMP Assay for IDV

We evaluated the primer sets generated for the PB1 and PB2 gene sequences of IDV in silico for their end stabilities, formation of hairpins, self-dimers, and heterodimers. We found that none of the sets targeting the PB1 gene passed our filters, whereas 2 primer sets targeting the PB2 gene met our criteria and were synthetized (Appendix Table 2). 

We initially trialed both primer sets by performing the RT-LAMP reaction in a real-time thermocycler targeting gBlocks containing the complete PB2 sequence of IDV, as well as IAV, IBV, and ICV as negative controls, using SYTO9 DNA dye to track the accumulation of amplification products over time (Figure 1). Each gBlock was incubated at a concentration of 10^6^ copies per µL of input. Because nonspecific amplification is a well-known problem with RT-LAMP reactions, we incubated the reactions for <80 minutes to assess the maximum incubation time for each primer set, defined as the longest time in which reactions could proceed without producing false-positive signals in the negative samples or nontemplate controls. Although both primer sets amplified the IDV target gBlock before the 20-minute mark, the IDV_set1 showed early onset of nonspecific amplification at ≈40 minutes (Figure 1, panel A), compared with 65 minutes with the IDV_set2 (Figure 1, panel B). Because of the increased specificity, we chose IDV_set2 for all subsequent analysis and defined the maximum incubation time as 60 minutes. We defined the threshold at the start of the exponential phase of amplification.

**Figure 1:**
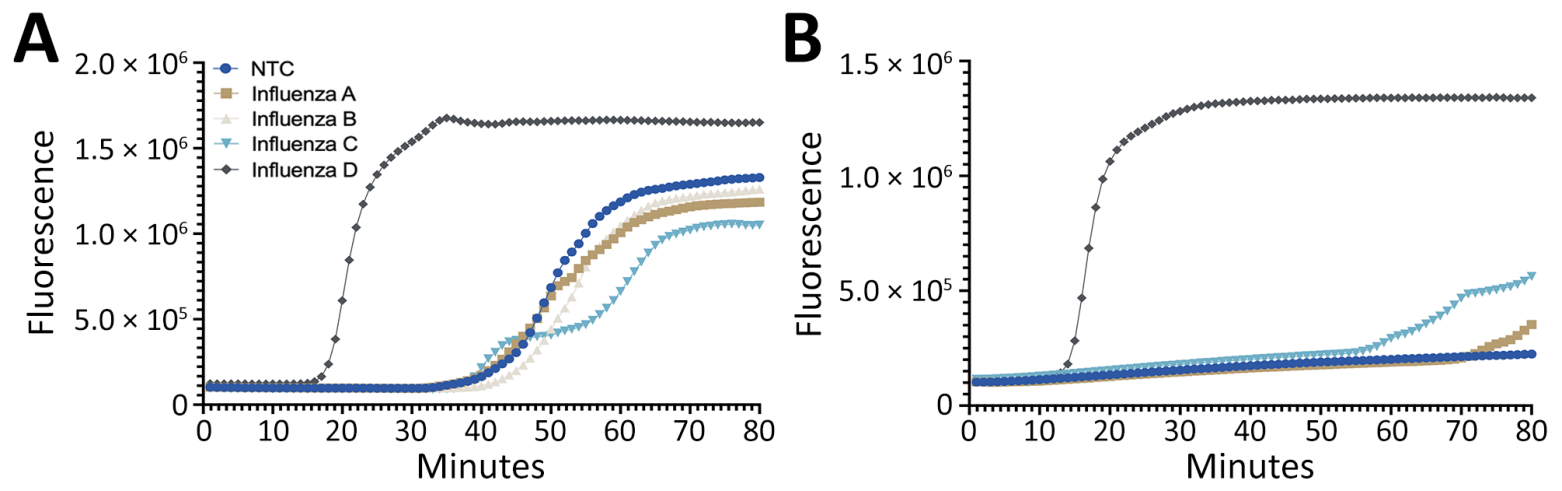
Mean amplification curves from reverse transcription loop-mediated isothermal amplification (RT-LAMP) reaction triplicates showing the onset of specific and nonspecific signal over time in study of isothermal detection of influenza D virus (IDV) using RT-LAMP. Graphs indicate results of RT-LAMP on gBlocks containing the influenza A, B, C, and D virus polymer basic protein 2 gene sequences. All gBlocks were tested at a concentration of 10^6^ copies per µL of input. A) Reactions using the primers in IDV_set1. The amplification of the IDV gBlock positive control started within the first 20 minutes, whereas influenza A, influenza B, and influenza C gBlocks and the NTC (water) produced false-positive signals after only 35 minutes of incubation. B) Reactions using the primers in IDV_set2. The amplification of the IDV gBlock positive control started within the first 15 minutes, whereas influenza A, influenza B, and influenza C gBlocks and the NTC produced delayed false-positive signals after 65 minutes of incubation. NTC, nontemplate control.

To ensure complete coverage of >90% of the published IDV lineages, including the 3 viral isolates we had access to (D/bovine/France/5920/2014, D/swine/Oklahoma/1334/2011, and D/swine/England/126471/2023), we incorporated degenerate nucleotides into the primers from IDV_set2 where necessary (Figure 2; Appendix Table 2). To test whether the primer set containing degenerate nucleotides enabled detection of all 3 available isolates of IDV, we incubated dilutions of extracted RNA at 10^4^ and 10^3^ copies/µL for each of the isolates. All concentrations of the 3 isolates were efficiently amplified after ≈15 minutes (Figure 3).

**Figure 2:**
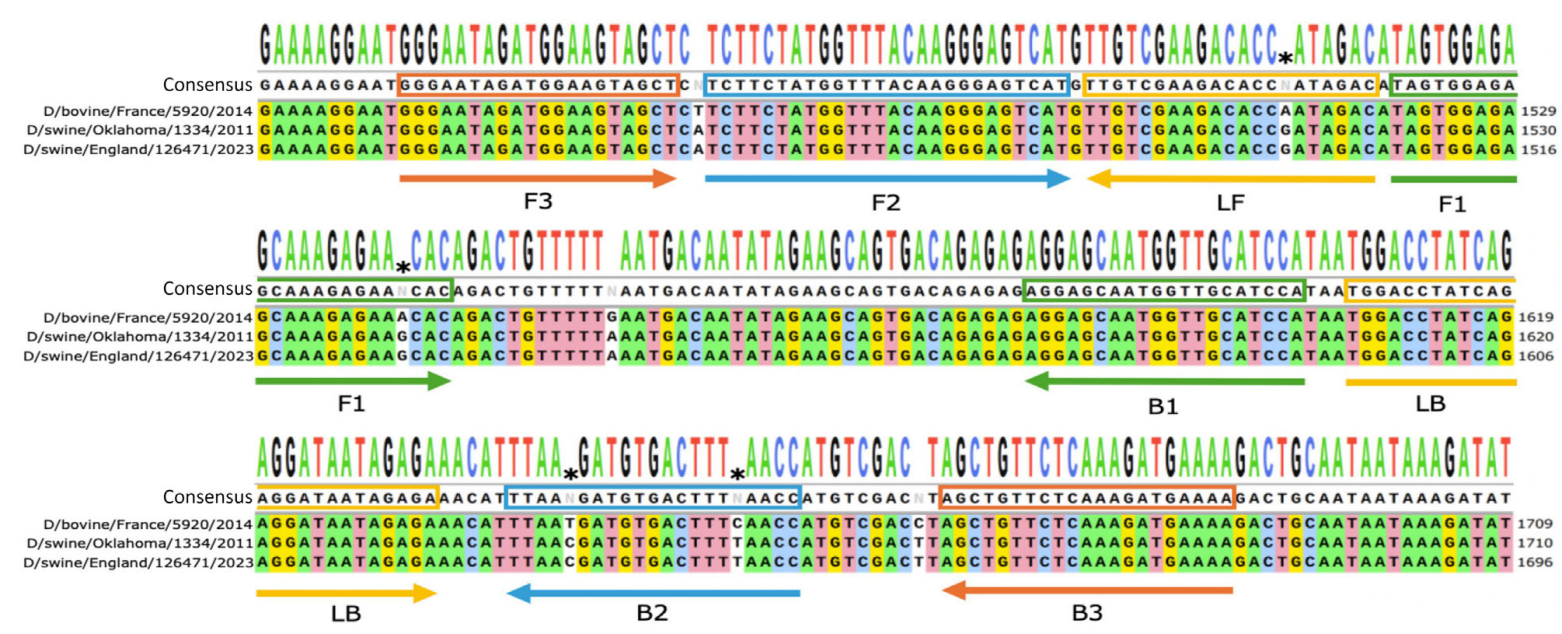
Reverse transcription loop-mediated isothermal amplification (RT-LAMP) primers targeting the influenza D virus polymer basic protein 2 gene in study of isothermal detection of influenza D virus. All available influenza D virus polymer basic protein 2 gene sequences in the National Center for Biotechnology Information Nucleotide database (https://www.ncbi.nlm.nih.gov/nucleotide) were aligned and mutations were annotated. To improve coverage, primers were synthesized with degenerate nucleotides in positions with heterogeneity in the sequences. The consensus sequence and sequences of the 3 isolates (D/bovine/France/5920/2014, D/swine/Oklahoma/1334/2011, and D/swine/England/126471/2023) are shown for comparison. Primer target sequences are presented within the boxes, and the direction of binding is represented by the arrows.

**Figure 3:**
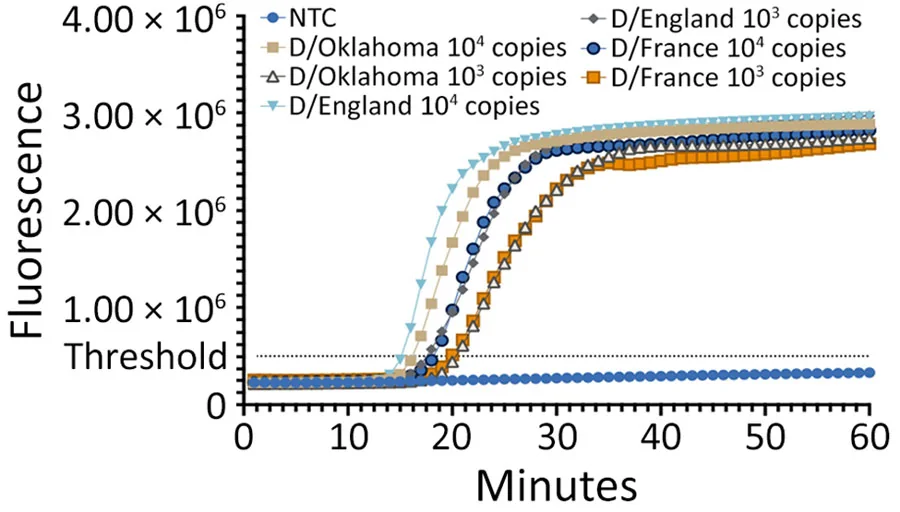
Mean amplification curves from reverse transcription loop-mediated isothermal amplification reaction triplicates showing the onset of amplification in study of isothermal detection of influenza D virus. All influenza D virus isolate RNAs at 10^4^ and 10^3^ copies per µL amplified within the first 20 minutes regardless of the lineage. NTC, nontemplate control.

### High Sensitivity and Specificity in Colorimetric RT-LAMP Assay

Having demonstrated that the RT-LAMP primers efficiently detected IDV RNA using a real-time thermocycler, we incubated subsequent reactions in a conventional thermocycler and analyzed the results visually on the basis of a colorimetric change in the reaction from pink (negative) to yellow (positive). To demonstrate that the addition of the degenerate nucleotides did not affect primer specificity, we incubated a high number of gBlock copies (10^6^ copies/µL) of the PB2 gene of influenza A, B, C, and D with the RT-LAMP mixture for 60 minutes at 65°C. Only the IDV target was amplified (Figure 4, panel A), suggesting that our assay is specific to IDV. This result was confirmed through gel electrophoresis of the amplicons (Figure 4, panel B).

**Figure 4:**
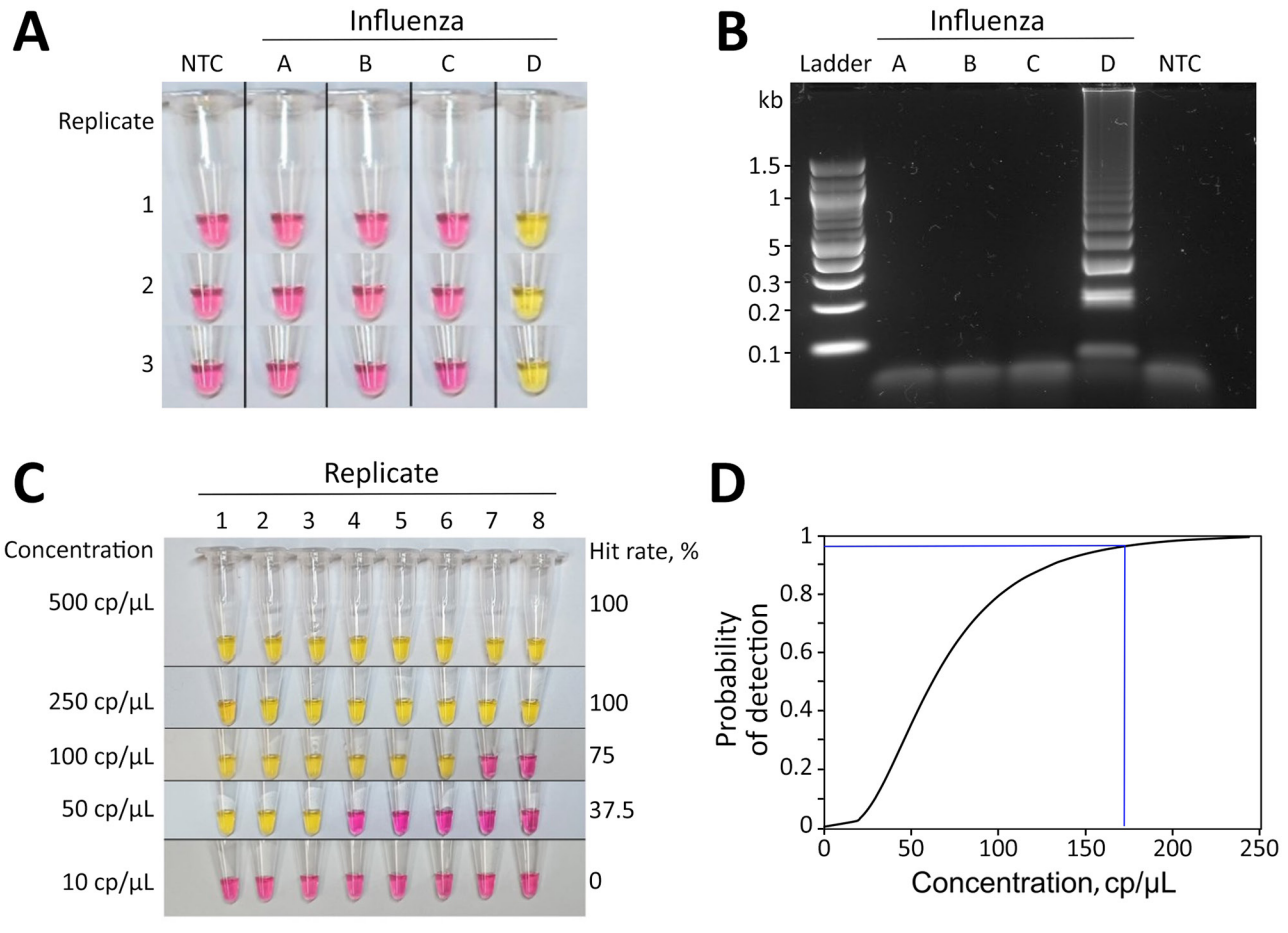
Specificity and sensitivity assays for influenza D virus (IDV) detection in study of isothermal detection of IDV using reverse transcription loop-mediated isothermal amplification (RT-LAMP). A) The total of 10^6^ copies/µL of gBlocks containing the influenza A, B, C and D virus polymer basic protein 2 gene sequences were tested. Only the gBlocks containing the target sequences were amplified, as demonstrated by color change in reactions. B) Two percent gel electrophoresis of 1 representative of each amplification products of the RT-LAMP reactions shown in panel A, showing the typical lamp ladder-like pattern in IDV virus samples and no visible nonspecific bands in the other target samples. C) Dilutions of target RNA in 8 replicates for sensitivity analysis at different concentrations, varying from 500 copies per µL (cp/μL) of target (top row) to 10 cp/μL of target (bottom row). D) Probit-analysis curve of probability of detection in relation to the number of copies. The limit of detection is defined as the minimum number of copies detected >95% of the time. NTC, nontemplate control.

Next, we estimated the sensitivity of the assay from the amplification results of 8 replicates at low target concentrations (500, 250, 100, 50, and 10 copies per µL of input) using an equimolar mix containing the RNA from the 3 IDV isolates used in this study. We plotted the calculated probit regression curve; the minimum number of copies detected 95% of the time was estimated to be 167 copies/µL (95% CI 103–3115 copies/µL) (Figure 4, panels C and D).

### Evaluation of RT-LAMP Assay on Spiked and Diagnostic Samples

To assess the efficiency of the RT-LAMP test in a more complex matrix, we spiked varying concentrations of IDV RNA into total RNA extracted from swine lung tissue. Before the addition of the IDV RNA, the swine lung tissue was confirmed to be free of IDV RNA by qRT-PCR. We tested 27 spiked samples in duplicate in different amounts of swine lung RNA to mimic different target/background ratios, using 4 nonspiked samples as negative controls (a total of 58 individual RT-LAMP reactions). We also quantified the IDV RNA in all spiked samples by qRT-PCR. The RT-LAMP assay successfully detected all 30 reactions that had a sample Cq <29. It failed to amplify 2 out of 6 reactions using samples with a Cq >29 to <30, 4 out of 14 reactions using samples with Cq >30 to <32, and 3 out of 4 reactions using samples with a Cq >32 (Figure 5, panel A). All 4 uninfected swine lung RNA samples were negative.

**Figure 5:**
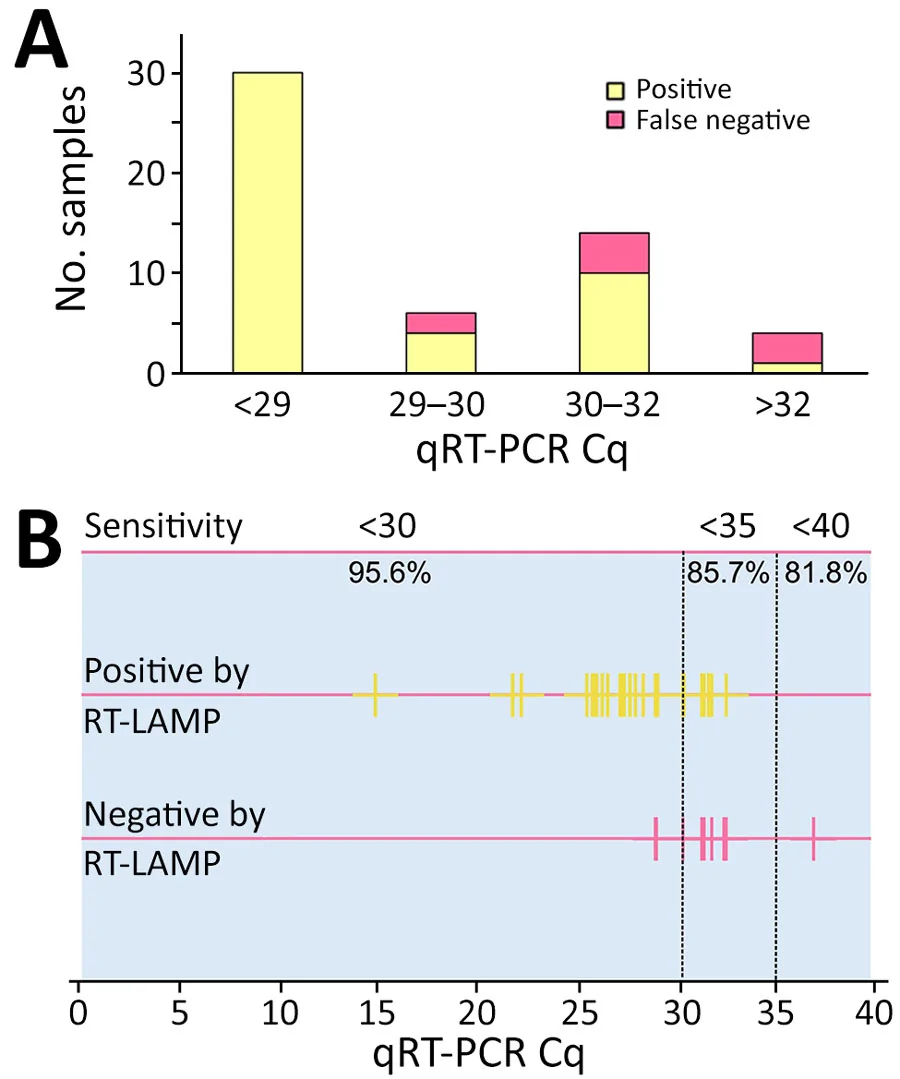
Sensitivity of RT-LAMP assay in relation to qRT-PCR Cq in study of isothermal detection of influenza D virus using RT-LAMP. A) Detection of spiked samples detected by RT-LAMP in relation to the Cq value. B) Each true positive sample was sorted according to its Cq value and RT-LAMP result. Yellow lines along the top line were positive by both RT-LAMP and qRT-PCR, pink lines on the lower line were positive by qRT-PCR but negative by RT-LAMP. The sensitivity of each range is presented as percentages at the top of the graph. Cq, cycle quantification; RT-LAMP, reverse transcription loop-mediated isothermal amplification; qRT-PCR, quantitative reverse transcription PCR.

Finally, we tested the performance of the RT-LAMP assay in detecting IDV viral RNA in total RNA extracted from samples collected from animals in the field. We analyzed a total of 46 RNA samples extracted from cattle and swine. The samples were composed of negative samples, samples containing IDV, and samples containing IAV RNA. We tested the samples in triplicate and compared the results with qRT-PCR results (Table). The RT-LAMP assay successfully detected IDV RNA in 3 of the 4 animal samples that were positive for IDV RNA by qRT-PCR.

**Table:** Comparison between qRT-PCR and RT-LAMP results for animal RNA samples in study of isothermal detection of influenza D virus using RT-LAMP*

Sample	IDV RT-LAMP	IDV qRT-PCR	IAV qRT-PCR
1	Negative	Negative	Negative
2	Negative	Negative	Positive, Cq 36.45
3	Negative	Negative	Negative
4	Negative	Negative	Negative
5	Negative	Negative	Negative
6	Negative	Negative	Negative
7	Negative	Negative	Negative
8	Negative	Negative	Positive, Cq 39.24
9	Negative	Negative	Negative
10	Negative	Negative	Negative
11	Negative	Negative	Negative
12	Negative	Negative	Negative
13	Negative	Negative	Negative
14	Negative	Negative	Positive, Cq 32.19
15	Negative	Negative	Negative
16	Positive	Positive, Cq 22.29	Negative
17	Negative	Negative	Negative
18	Negative	Negative	Negative
19	Negative	Negative	Negative
20	Negative	Negative	Positive, Cq 27.88
21	Negative	Negative	Negative
22	Negative	Negative	Negative
23	Negative	Negative	Negative
24	Negative	Positive, Cq 37.09	Negative
25	Negative	Negative	Negative
26	Negative	Negative	Negative
27	Negative	Negative	Negative
28	Negative	Negative	Negative
29	Negative	Negative	Negative
30	Negative	Negative	Negative
31	Negative	Negative	Negative
32	Negative	Negative	Negative
33	Negative	Negative	Negative
34	Negative	Negative	Negative
35	Positive	Positive, Cq 14.84	Negative
36	Negative	Negative	Negative
37	Negative	Negative	Negative
38	Positive	Positive, Cq 21.81	Negative
39	Negative	Negative	Negative
40	Negative†	Negative	Negative
41	Negative	Negative	Negative
42	Negative	Negative	Negative
43	Negative	Negative	Negative
44	Negative	Negative	Negative
45	Negative	Negative	Negative
46	Negative	Negative	Negative

*Cq, cycle quantification; IAV, influenza A virus; IDV, influenza D virus; qRT-PCR, quantitative reverse transcription PCR; RT-LAMP, reverse transcription loop-mediated isothermal amplification. †Sample number 40 showed a change in color in 1 replicate that did not fully convert into yellow, but for statistical calculations, it was considered a  false positive.

Altogether, we tested a total of 196 individual RT-LAMP reactions, consisting of 58 spiked RNA and 138 animal RNA samples. We compared the RT-LAMP results to the results of the standard qRT-PCR and calculated the sensitivity, specificity, positive and negative likelihood ratios, and positive and negative predictive values. Given that amplification of low abundance targets close to the limit of detection are probabilistic events, multiple replicates are required to estimate the detection probability (or hit rate) at a given concentration. To account for that, we treated every replicate of a spiked and diagnostic sample as an independent observation for statistical analysis, and we compared each result individually with qRT-PCR. A total of 54 of reactions were positive both by qRT-PCR and RT-LAMP (true positive), 129 were negative by both qRT-PCR and RT-LAMP (true negative), 12 were positive by qRT-PCR and negative by RT-LAMP (false negative), and 1 was positive by RT-LAMP but negative by qRT-PCR (false positive) (Appendix Table 3). The calculated specificity of the test was 99.23%, the sensitivity of the test was 81.82%, and the overall accuracy was 93.37%. The sensitivity of the test for samples with a Cq <35 was 85.7%; sensitivity for samples with a Cq <30 was 95.6% (Figure 5, panel B; Appendix Table 4).

## Discussion

We developed a colorimetric RT-LAMP assay capable of detecting IDV RNA in both experimentally spiked and field-collected samples. The assay demonstrated a high analytical specificity of 99.23%, as well as analytical sensitivity of 167 viral RNA copies/µL of target input within a 60-minute reaction time, using a 1-step assay and a simple colorimetric detection system for result interpretation. The single-tube format enables simpler handling because fewer manipulations are required, decreasing time from collection to final result. Active viral infections typically produce millions of viral copies; therefore, the detection limit in the hundreds of copies range (equivalent to Cq values >30 in qRT-PCR) will enable infections to be identified during early-stage viral replication and throughout extended viral shedding periods. Although sensitivity remains lower than qRT-PCR methodologies, the assay exceeds the analytical performance of most rapid antigen tests and could provide direct evidence of active infection compared with serologic approaches.

Primer design incorporated strategically positioned degenerate nucleotides to ensure comprehensive viral lineage coverage, achieving detection capability for >90% of published IDV sequences, including the recently identified D/swine/England/126471/2023 lineage from UK swine populations (*20*). The degenerate nucleotides were specifically integrated avoiding the ends of the primers to minimize potential amplification interference while maximizing lineage inclusivity.

Current IDV detection relies mainly on qRT-PCR and antibody detection, which both present distinct analytical trade-offs that affect surveillance implementation strategies. qRT-PCR is the standard method and offers superior analytical sensitivity with viral load quantification, enabling precise molecular characterization essential for genetic surveillance and outbreak investigation. However, that approach requires sophisticated laboratory infrastructure, specialized personnel, and complex sample preparation protocols. Those requirements limit point-of-care applications and increase costs per sample. Conversely, antibody sampling provides simplified collection protocols with enhanced sample stability and cost-effective population seroprevalence assessment, enabling large-scale epidemiologic studies. Nevertheless, serologic methods exhibit inherent limitations because of seroconversion kinetics, cannot distinguish active from historical infections, and might demonstrate cross-reactivity with related viruses, precluding their utility for acute infection diagnosis and real-time outbreak response. Those complementary yet functionally distinct methodological constraints necessitate the development of alternative diagnostic platforms that bridge the gap between laboratory-based precision and field-deployable practicality.

RT-LAMP offers a promising point-of-care alternative to qRT-PCR, because its simplified equipment requirements and minimal reaction components enable field-deployable testing with reduced sample processing demands. The colorimetric RT-LAMP approach provides qualitative detection results, enabling rapid screening of larger sample volumes at lower per-reaction costs than conventional qRT-PCR methodologies. Even though RT-LAMP might be used to differentiate between known lineages, RT-LAMP cannot fully replace qRT-PCR or sequencing for comprehensive genetic surveillance applications, because its amplification products are difficult to sequence. That issue is particularly problematic when detecting novel or uncharacterized viral lineages that have not been sequenced and deposited in public databases. Despite those genetic surveillance limitations, RT-LAMP offers potential for cost-effective sample triage protocols, enabling preliminary screening before confirmatory molecular characterization through more sophisticated diagnostic platforms.

In summary, our RT-LAMP assay for detecting IDV demonstrated high specificity and sensitivity, indicating its utility as a rapid and accessible diagnostic tool. To further enhance its applicability, future optimizations could focus on integrating the assay into lab-on-a-chip microfluidic platforms or coupling the reaction with a compact detector, enabling fully automated, portable testing. Further field validation across varied sample types and conditions will be essential; however, embedding this RT-LAMP assay into existing surveillance infrastructures can bolster real-time IDV detection and strengthen One Health strategies for pandemic preparedness.

Appendix Additional information about isothermal detection of influenza D virus using reverse transcription loop-mediated isothermal amplification.
