# Supplementary material for: Detection of Influenza D Virus using Reverse Transcription Loop-Mediated Isothermal Amplification
Source: Emerg Infect Dis. 2026 Oct;32(10):1636–44. doi: 10.3201/eid3210.260499 (PMC13632844; doi:10.3201/eid3210.260499)
Supplement: Supplementary file 1 [file 26-0499-Techapp-s1.pdf]

Article DOI: https://doi.org/10.3201/eid3210.260499

EID cannot ensure accessibility for supplementary materials supplied by authors. Readers who have difficulty accessing supplementary content should contact the authors for assistance.

Isothermal Detection of Influenza D Virus using Reverse Transcription Loop-Mediated Isothermal Amplification

Appendix

Appendix Table 1. Influenza D PB1 and PB2 genes accession numbers used for primer design.

Genes	Accession numbers
PB1	MN123875.1, LC318666.1, JQ922306.1, KF425660.1, KF425653.1, KM015506.1, KM015499.1, KM015492.1, KM392504.1, KM392497.1, KM392490.1, KM392483.1, KM392476.1, KM392469.1, KT592521.1, KT592531.1, KT581410.1, KU710435.1, LC128438.1, KX768825.1, KX768832.1, KX768839.1, LC270266.1, NC_036615.1, LC522352.1, MT636471.1, MW020320.1, MW632179.1, MW632172.1, MW632186.1, MZ264978.1, KU710434.1, KU710436.1, KU710437.1, MK131034.1, MN165183.1, MN165190.1, MN165197.1, MN165204.1, MN165211.1, MN165218.1, MN165225.1, MN165238.1, MN165245.1, MN165252.1, MN123958.1, MN123917.1, MN123889.1, MN123924.1, MN123896.1, MN123938.1, MN123945.1, MN123910.1, MN123952.1, MT349511.1, MT349518.1, MT349525.1, MT349537.1, MT349547.1, MT349559.1, MT349566.1, MT349573.1, MT349580.1, MT349601.1, MT349608.1, MT349622.1, MT349636.1, MT349642.1, MT349649.1, MN123868.1, MT349532.1, MW079437.1, MW079438.1, MW079440.1, MW079441.1, MW079442.1, KT592524.2, MW020313.1, MW079439.1, MW079443.1, MN165232.1, MW020306.1, MT349615.1, MK965363.1, MK965364.1, MK965366.1, MK965367.1, MK965368.1, MK965369.1, MK965370.1, MK965372.1, OP020133.1, MK965365.1, MK131023.1, OM468206.1, OM468207.1, OM468208.1, OM468209.1, OM468210.1, OM468211.1, OM468212.1, OM468213.1, OM468214.1, OM468215.1, OM468216.1, MK101119.1, MN123882.1, MT246284.1, KF425667.1, MK054182.1, LC494106.1, MT511334.1, MT511341.1, MT511348.1, MT511355.1, MT511362.1, MT511369.1, MT511375.1, MT511382.1, MT511389.1, MT511396.1, MT511403.1, MT511410.1, MT511417.1, MT511424.1, MT511431.1, MT511438.1, MT511445.1, MT511451.1, MT511458.1, MT511465.1, MT511472.1, MT511479.1, MT511486.1, MT511493.1, MT511500.1, MT511507.1, MT511513.1, MT511520.1, MT511527.1, MT511534.1, MT511541.1, OM763675.1, ON038815.1, ON038816.1, ON038817.1, ON038818.1, ON038819.1, ON038820.1, ON038821.1, ON038822.1, ON038823.1, ON038824.1, ON038825.1, ON038826.1, ON038827.1, ON038828.1, ON038829.1, ON038830.1, ON038831.1, ON038832.1, ON038833.1, ON038834.1, ON038835.1, MG720241.1, MT246271.1
PB2	JQ922305.1, KF425652.1, KF425659.1, KF425666.1, KM015491.1, KM015498.1, KM015505.1, KM392468.1, KM392475.1, KM392482.1, KM392489.1, KM392496.1, KM392503.1, KT581409.1, KT592516.1, KT592523.1, KT592530.1, KU710438.1, KU710439.1, KU710440.1, KU710441.1, KX768817.1, KX768824.1, KX768831.1, KX768838.1, LC128439.1, LC270265.1, LC318665.1, LC494105.1, LC522351.1, LC565467.1, LC565468.1, LC565469.1, LN559120.1, MG720239.1, MK054181.1, MK101118.1, MK131022.1, MK131029.1, MK965373.1, MK965374.1, MK965375.1, MK965376.1, MK965377.1, MK965378.1, MK965379.1, MK965380.1, MK965381.1, MK965382.1, MK965383.1, MK965384.1, MK965385.1, MK965386.1, MK965387.1, MK965388.1, MN123862.1, MN123863.1, MN123876.1, MN123877.1, MN123884.1, MN123891.1, MN123898.1, MN123905.1, MN123912.1, MN123919.1, MN123933.1, MN123940.1, MN123947.1, MN165175.1, MN165182.1, MN165189.1, MN165196.1, MN165203.1, MN165210.1, MN165217.1, MN165224.1, MN165231.1, MN165237.1, MN165244.1, MN165251.1, MN173616.1, MT246272.1, MT246279.1, MT246285.1, MT246292.1, MT246299.1, MT349510.1, MT349517.1, MT349524.1, MT349531.1, MT349536.1, MT349546.1, MT349554.1, MT349565.1, MT349572.1, MT349579.1, MT349586.1, MT349593.1, MT349600.1, MT349607.1, MT349614.1, MT349621.1, MT349628.1, MT349635.1, MT349641.1, MT349648.1, MT511333.1, MT511340.1, MT511347.1, MT511354.1, MT511361.1, MT511368.1, MT511374.1, MT511381.1, MT511388.1, MT511395.1, MT511402.1, MT511409.1, MT511416.1, MT511423.1, MT511430.1, MT511437.1, MT511444.1, MT511450.1, MT511457.1, MT511464.1, MT511471.1, MT511478.1, MT511485.1, MT511492.1, MT511499.1, MT511506.1, MT511512.1, MT511519.1, MT511526.1, MT511533.1, MT511540.1, MT636470.1, MW020305.1, MW020312.1, MW020319.1, MW079430.1, MW079431.1, MW079432.1, MW079433.1, MW079434.1, MW079435.1, MW079436.1, MW632171.1, MW632178.1, MW632185.1, MZ264973.1, NC_036616.1, OK513022.1, OM468185.1, OM468186.1, OM468187.1, OM468188.1, OM468189.1, OM468190.1, OM468191.1, OM468192.1, OM468193.1, OM468194.1, OM468195.1, OM468196.1, OM468197.1, OM468198.1, OM468199.1, OM468200.1, OM763674.1, ON038795.1, ON038796.1, ON038797.1, ON038798.1, ON038799.1, ON038800.1, ON038801.1, ON038802.1, ON038803.1, ON038804.1, ON038805.1, ON038806.1, ON038807.1, ON038808.1, ON038809.1, ON038810.1, ON038811.1, ON038812.1, ON038813.1, ON038814.1, ON038920.1, ON415263.1, OP020132.1, OP474071.1

Appendix Table 2. RT-LAMP primers for the detection of Influenza D. Degenerate nucleotides are shown in bold. R: A or G; Y: C or T.

Primer Name	Sequence (5′ – 3′)
IDV_set1.F3	TGCCTTGTTGAGTACAAGAG
IDV_set1.B3	ATAGGTCCATTATGGATGCA
IDV_set1.FIP	ACTCCCTTGTAAACCATAGAAGATGTTTTTTTGCTCATATTGGACAATTCGG
IDV_set1.BIP	CGATAGACATAGTGGAGAGCAAAGTTTTTTCCATTGCTCCTCTCTCTGT
IDV_set1.LF	TCCATCTATTCCCATTCCTT
IDV_set1.LB	AGAAGCACAGACTGTTT
IDV_set2.F3	GGGAATAGATGGAAGTAGCT
IDV_set2.B3	TTTTCATCTTTGAGAACAGCT
IDV_set2.FIP	GTG**Y**TTCTCTTTGCTCTCCACTATTTTTTTTGGTTTACAAGGGAGTCAT
IDV_set2.BIP	AGGAGCAATGGTTGCATCCATTTTTTTGGTT**R**AAAGTCACATC**R**TTAA
IDV_set2.LF	GTCTAT**Y**GGTGTCTTCGACAA
IDV_set2.LB	TGGACCTATCAGAGGATAATAGAGA

Appendix Table 3. Total number of reactions accounted for statistical analysis. Only the reactions with spiked and animal collected RNA were considered for these calculations.

	RT-qPCR				
RT-LAMP	Positive	n	Negative	n	Total
Positive	True Positive	54	False Positive	1	55
Negative	False Negative	12	True Negative	129	141
Total		66		130	196

True positive: Positive both by RT-LAMP and RT-qPCR. True Negative: Negative both by RT-LAMP and RT-qPCR. False Negative: Negative by RT-LAMP and positive by RT-qPCR. False Positive: Positive by RT-LAMP and negative by RT-qPCR.

Appendix Table 4. Complete statistical analysis results of the data presented in Appendix Table 2. The sensitivity is the probability the test will be positive when the viral RNA is present

Statistic	Value	95% CI
Sensitivity	81.82%	70.39% to 90.24%
Specificity	99.23%	95.79% to 99.98%
Disease prevalence *	33.67%	27.10% to 40.75%
Positive Predictive Value	98.18%	88.42% to 99.74%
Negative Predictive Value	91.49%	86.56% to 94.72%
Accuracy	93.37%	88.93% to 96.42%

The specificity is the probability the test will be negative when the viral RNA is not present. The positive predictive value is the probability the viral RNA is present if the test is positive, and the negative predictive value is the probability the viral RNA is not present if the test is negative. The accuracy of the test is defined as the overall probability samples are correctly classified. *The disease prevalence refers only to the sample size and not the actual prevalence of the disease.
